# Gender differences in cancer risk after kidney transplantation

**DOI:** 10.18632/oncotarget.26859

**Published:** 2019-05-03

**Authors:** Anna Buxeda, Dolores Redondo-Pachón, María José Pérez-Sáez, Álvaro Bartolomé, Marisa Mir, Ana Pascual-Dapena, Anna Sans, Xavier Duran, Marta Crespo, Julio Pascual

**Affiliations:** ^1^ Department of Nephrology, Hospital del Mar, Barcelona, Spain; ^2^ Department of Medicine, University Autonoma Barcelona, Barcelona, Spain; ^3^ Department of Experimental and Health Sciences, University Pompeu-Fabra, Barcelona, Spain; ^4^ Methodology and Biostatistics Support Unit, Institute Hospital del Mar for Medical Research (IMIM), Barcelona, Spain

**Keywords:** cancer, immunosuppression, kidney transplant, gender, risk factor

## Abstract

Kidney transplant (KT) recipients are at greater risk of developing some cancers than the general population. Moreover, cancer is the only cause of death that is currently increasing after kidney transplantation. We analyzed incidence, risk factors and characteristics of post-transplant malignancies (solid organ tumors and lymphoproliferative disorders) at our center in 925 KT recipients (1979-2014). Sex differences were particularly assessed.

One hundred and eight patients (11.7%) developed solid organ tumors (76.9%) or lymphoma (23.1%). Twenty-one percent of patients who reached 20 years after KT developed cancer, with a median post-KT time to diagnosis of 7.4 years. Most common solid organs affected were lung (30.1%), prostate (10.8%), bladder (9.6%), and native kidney (7.2%). When analyzing standardized incidence ratios (SIR) by gender compared to the general population, relative risk was increased in women (SIR = 1.81; 95%CI, 1.28–2.45) but not significantly increased in men (SIR = 1.22; 0.95–2.52). Regarding specific types, gynecological (SIR = 11.6; 4.2–22.7) and lung (SIR = 10.0; 4.3–18.2) in women, and bladder (SIR = 16.3; 5.9–32.1) in men were the most affected locations. Thymoglobulin, a polyclonal antibody that has been used as an immunosuppressive agent in kidney transplantation over the last decades, was a significant risk factor for developing cancer in adjusted regression analysis [IRR = 1.62, 1.02–2.57; p = 0.041], and was associated with lower patient survival.

Compared with the general population, the incidence of post-KT non-skin cancer is almost two-fold higher in women but not significantly higher in men. Lung is the most common solid organ affected. Thymoglobulin induction therapy is associated with a greater risk.

## INTRODUCTION

In patients with end-stage kidney disease (ESKD) and requirement of renal replacement therapy, kidney transplantation (KT) is preferred over dialysis as it provides both improved patient survival and quality of life at lower costs [1–3]. However, an increased incidence of cancer has been reported in this population [4, 5]. Moreover, the outcomes and prognosis of KT patients with some cancers are substantially worse than in those patients with cancer but without kidney disease [6–8].

The cumulative incidence of *de novo* cancer after transplantation is 9–10% at 10 years and between 10–27% at 20 years, excluding non-melanoma skin cancer [3, 9–11]. The importance of cancer in KT relies on its impact on patient survival, being the second long-term cause of death in KT patients [12]. In fact, cancer after KT is the only cause of death that is currently increasing [13]. Immunosuppression causes a substantial increase in cancer risk [14, 15].

Age and male sex are recognized risk factors associated with the development of malignancy after KT [4]. Other factors have been described: underlying kidney disease, type of donor (deceased and expanded donor are at substantially increased risk of cancer compared with living donor) [16], cancer before transplant [17, 18], oncogenic viruses such as human papillomavirus (HPV) or Epstein-Barr virus (EBV) [19], acute rejection (AR) [20], race [21] or tobacco [22]. However, the literature on this subject is heterogeneous and except for cancers associated with lifestyle factors like tobacco or caused by viral infections, most of these risk factors have not been related to a specific cancer location.

In addition, overall immunosuppressive dose is associated with an increased risk of cancer following transplantation. Immunosuppression may facilitate carcinogenesis by decreasing mechanisms involved in the immunologic control of oncogenic viral infection and cancer immunosurveillance or by direct DNA damage [12]. Currently available immunosuppressive therapies influence different anti-cancer pathways, but the contributive effect of each agent is not well established at this moment [23]. T cell-depleting agents, such as thymoglobulin, are widely used as induction immunosuppressive therapy and to treat rejection in KT recipients. Earlier studies have demonstrated that the use of T cell-depleting antibodies is associated with an increased risk for post-transplantation lymphoproliferative disorders (PTLD) compared to interleukin-2 receptor alpha chain (IL-2Ra) agents or no induction therapy [24, 25]. On the other hand, mammalian target of rapamycin inhibitors (mTORi) have been reported to inhibit cancer progression in animals and to be associated with reduced incidence of posttransplant *de novo* malignancies in humans [26]. Nonetheless, authors have not been able to demonstrate improved survival in KT recipients taking mTORi [27].

The increased risk of post-KT malignancy is not spread evenly over all types of cancer [24]. Certain malignancies, such as lung, liver and kidney cancer, melanoma and non-melanoma skin cancer, PTLD, and thyroid cancer, are increased. Furthermore, risk is particularly high for malignancies caused by viral infections, including anogenital cancers (human papillomavirus), non-Hodgkin lymphoma and Hodgkin lymphoma (both due to EBV), Kaposi sarcoma (human herpes virus 8) and liver cancer (hepatitis C and B viruses) [4, 9, 14, 23].

Several population-registries have analyzed the incidence of cancer after KT in the United States [4], Australia and New Zealand [10], Europe [28] and Asia [29, 30]. Nevertheless, there is little evidence in the literature about incidence, risk factors and cancer location in southern European transplanted patients (mostly reported in Italian population) [31]. No data is available in Spainish renal transplant population, the country with the highest rate of KT per million population.

The aim of our study was to analyze the incidence of cancer in our cohort of KT recipients and compare it with general population, as well as to study the characteristics and risk factors of post-transplant malignancies (PTM). Donor characteristics, recipient’s medical history and immunosuppression regimens were evaluated. In particular, a gender effect was assessed.

## RESULTS

### Prevalence of cancer and distribution

**Figure 1:**
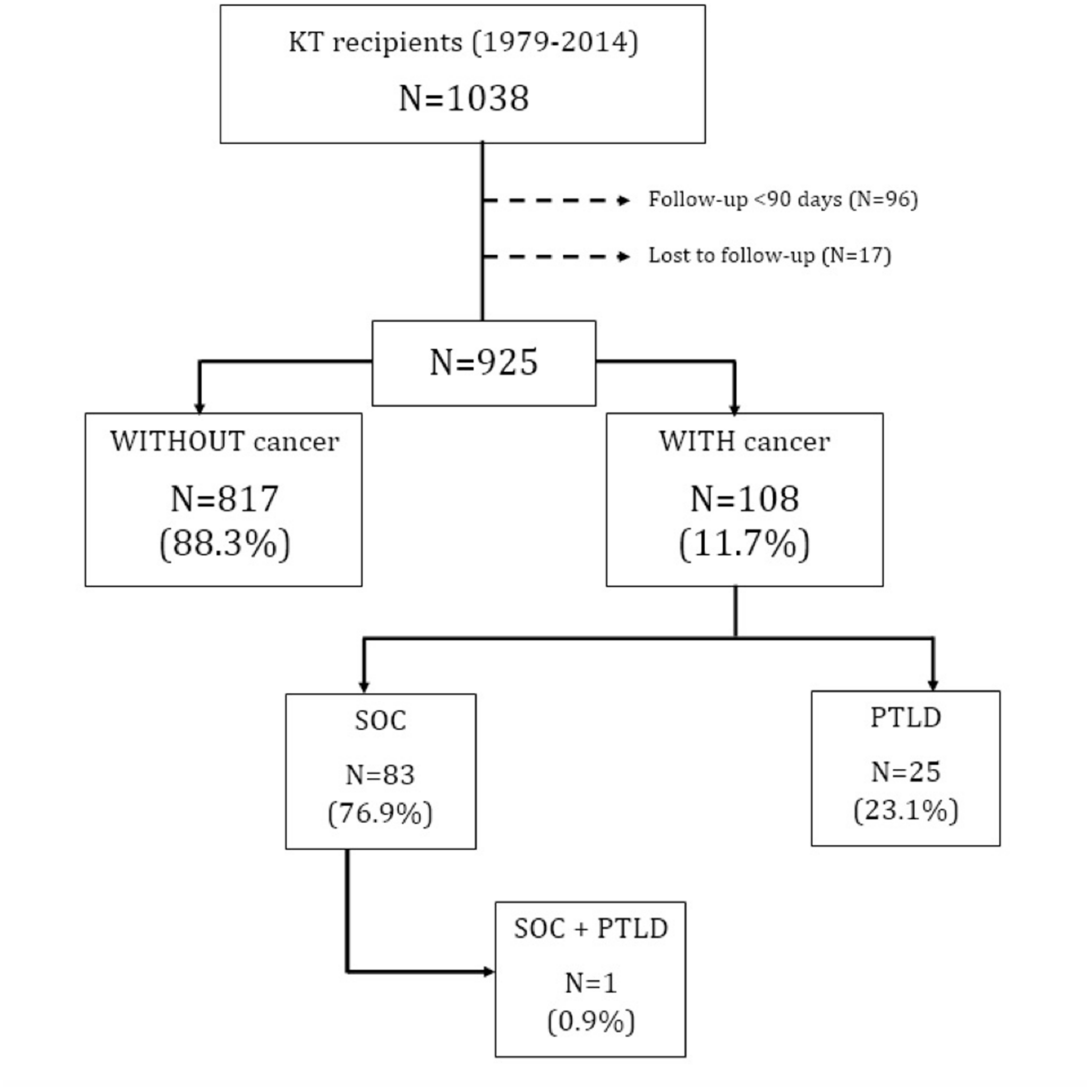
Patients flow-chart Abbreviations: KT: Kidney transplant, SOC: Solid organ cancer, PTLD: Post-transplantation lymphoproliferative disorders.

During the observation period (May 1979 to April 2016), from a total of 925 KT recipients, 108 (11.7%) developed at least one cancer. Eighty-three patients (76.8%) had at least one solid organ tumor, and 25 patients (23.1%) had a lymphoproliferative disorder. One patient had both entities (solid organ tumor and a lymphoproliferative disorder). (Figure 1).

**Figure 2:**
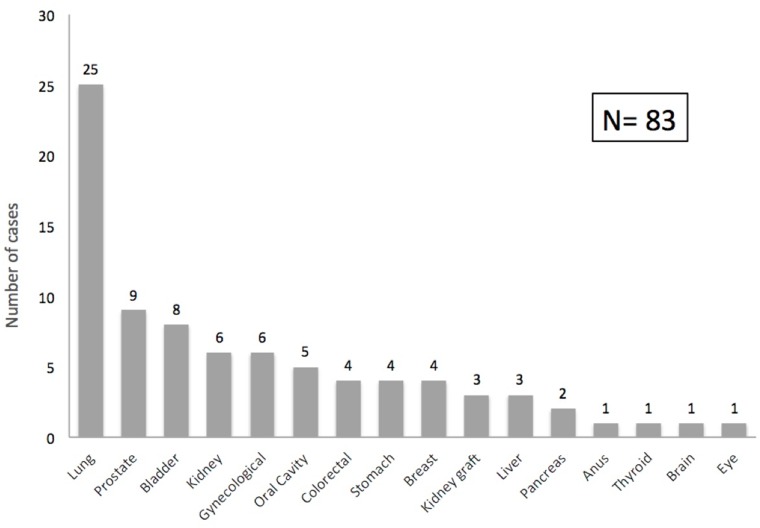
Post-transplant solid organ cancer distribution Graph shows the spectrum of malignancies after kidney transplantation (number of cases).

Lung cancer was the most frequently observed, accounting for 30.1% of all solid organ cancers in our cohort, followed by prostate (10.8%), urinary bladder (9.6%), native kidney (7.2%) and gynecological tumors (7.2%). The distribution of solid organ cancers is shown in Figure 2.

**Figure 3:**
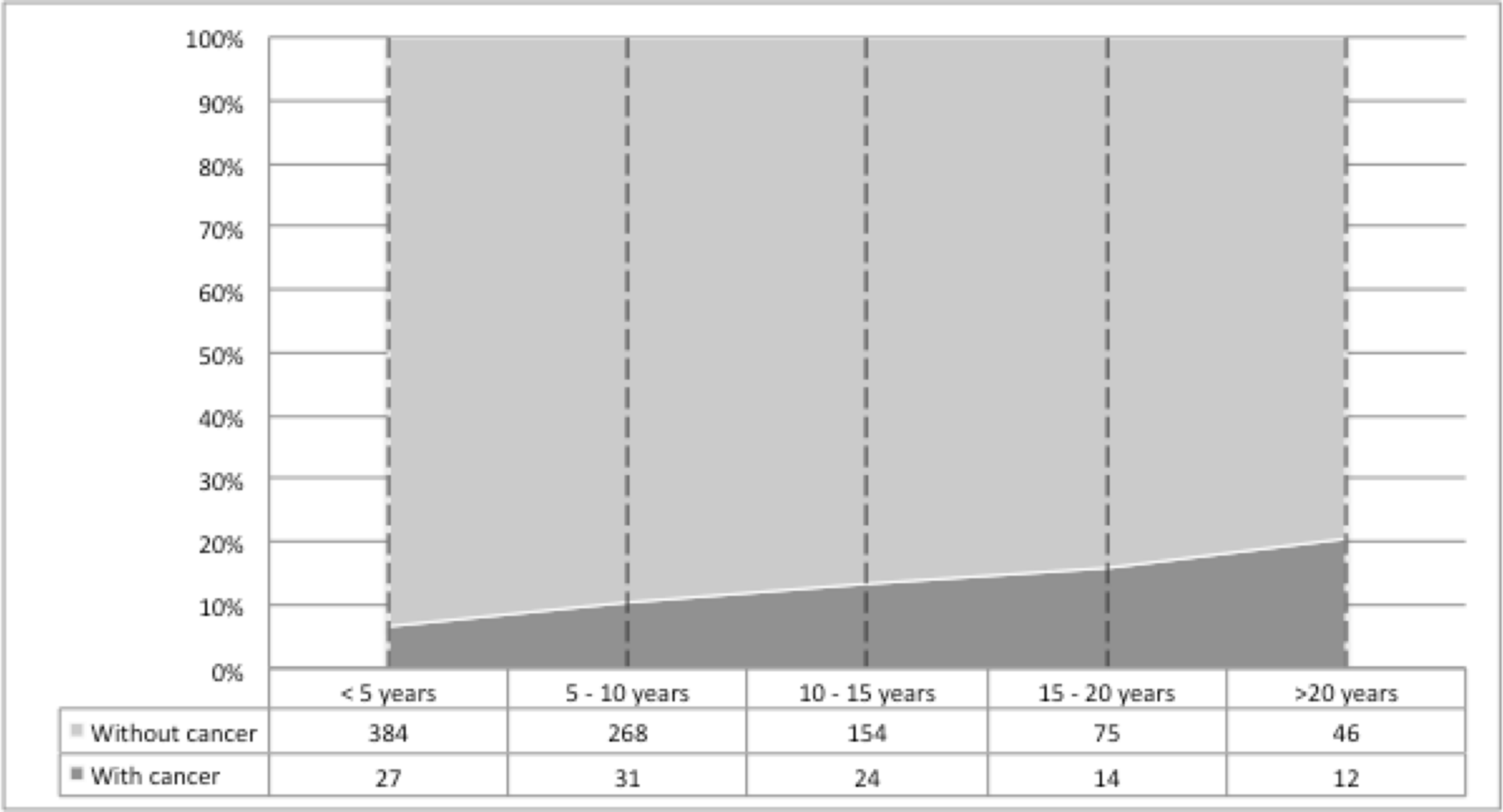
Cumulative incidence of post-transplant malignancies Cumulative incidence of post-transplant malignancies after kidney transplantation, according to time since transplant.

Median post-KT time to cancer diagnosis was 7.4 years (IQR 3.2-9.7), with an overall cumulative incidence of PTM at 20 years after transplantation of 20.7%. (Figure 3).

### Estimation of cancer risk in KT compared to the risk in the general population

**Figure 4:**
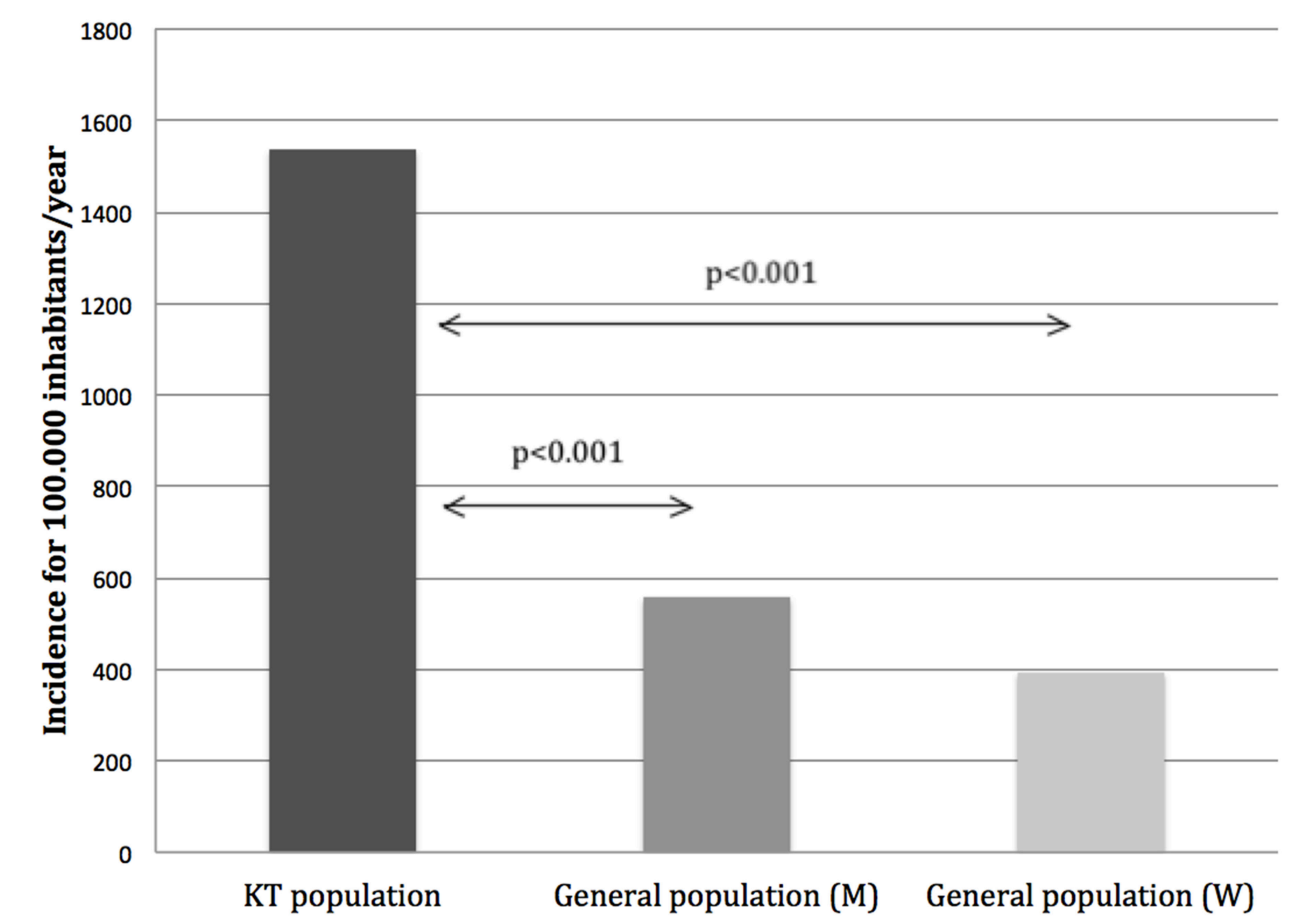
Estimated incidence of post-transplant malignancies (PTM) compared to general population in Catalonia Comparison of estimated incidence rates of PTM between our cohort (1536 cases per 100,000 inhabitants/year) and the general population in Catalonia (393.4 cases per 100,000 inhabitants/year for women and 557.6 cases per 100,000 inhabitants/year for men). Abbreviations: KT: Kidney transplant, M: Men, W: Women.

Based on these data, we determined that the estimated incidence rate of PTM in our cohort was 1536 cases per 100,000 inhabitants/year. This was higher than in general population in Catalonia, where the estimated incidence rate for malignancies (excluding non-melanoma skin cancer) is 393.4 cases per 100,000 inhabitants/year for women and 557.6 cases per 100,000 inhabitants/year for men [32] (Figure 4). Relative risks of non-skin cancers compared to the general population in Catalonia were expressed as age standardized incidence ratios (SIRs). When analyzing SIRs computed using sex of overall PTM compared to the general population in Catalonia, relative risk was increased in women (SIR = 1.81; 95% CI, 1.28–2.45) but not significantly increased in men (SIR = 1.22; 95% CI, 0.95–2.52). Nevertheless, in both sexes a wide range of malignancy types had more pronounced risk comparing with the general population. The locations with greatest risk were: gynecological (SIR = 11.59; 95% CI, 4.17–22.71), lung cancers (SIR = 10.05; 95% CI, 4.29–18.22) and PTLD (SIR = 5.95; 95% CI, 2.54–10.79) in women, and bladder (SIR = 16.35; 95% CI, 5.88–32.05), PTLD (SIR = 5.54; 95% CI, 3.22–8.49) and native kidney cancers (SIR = 4.48; 95% CI, 1.78–8.41) in men. On the contrary, the relative risk of malignancy types occurring most frequently in general population was only moderately elevated or not elevated, *e.g.,* breast, prostate and colorectal. (Table 1).

**Table 1:** Standardized incidence ratios by specific cancer types and gender

	Women		Men	
Organ Location	Number of cases at sample	SIR (95% CI)	Number of cases at sample	SIR (95% CI)
Lung	8	10.05 (4.29 - 18.22)	17	2.22 (1.29 - 3.4)
Bladder	2	5.76 (0.54 - 16.51)	6	16.35 (5.88 - 32.05)
PTLD	8	5.95 (2.54 - 10.79)	17	5.54 (3.22 - 8.49)
Gynecological	6	11.59 (4.17 - 22.71)	-	-
Kidney	2	4.98 (0.47 - 14.29)	7	4.48 (1.78 - 8.41)
Oral Cavity	2	8.24 (0.78 - 23.61)	3	1.6 (0.3 - 3.91)
Stomach	0	-	4	2.78 (0.72 - 6.18)
Colorectal	2	0.72 (0.07 - 2.05)	3	0.4 (0.08 - 0.98)
Liver	1	3.62 (0 - 14.19)	2	1.33 (0.13 - 3.82)
Pancreas	1	1.92 (0 - 7.52)	1	0.83 (0 - 3.25)
Breast	4	0.85 (0.22 - 1.88)	0	-
Prostate	-	-	9	0.77 (0.35 - 1.35)
Brain	0	-	1	1.48 (0 - 5.8)
Thyroid	1	2.86 (0 - 11.2)	0	-
Eye	0	-	1	0.06 (0 - 0.25)
All cancers	37	1.81 (1.28 - 2.45)	71	1.22 (0.95 - 1.52)

Standardized incidence ratios (SIRs) and 95% confidence intervals for most common de novo malignancies according to sex compared to the general population in Catalonia. Abbreviations: PTLD: Post-transplant lymphoproliferative disorder.

### Basal characteristics and risk factors

Baseline characteristics of recipients, donors, and KT are shown in Table 2. Mean recipient age at time of transplantation was 47.9 years (SD 14.2 years). The majority of recipients were male (62.8%) and Caucasian (93.3%). The most frequent primary renal disease was glomerulonephritis (n = 217, 24.4%), followed by polycystic kidney disease (n = 120, 13.5%). 87.7% of recipients were on hemodialysis before KT and, on average, they spent 22 months (IQR 11–41) on dialysis prior to KT. Kidney donors were predominantly male (57.9%), Caucasian (95.2%), and with a mean age of 46.9 (± 16.3) years-old. Induction immunosuppressive treatment was based on anti-CD25 drugs in 49.9% of cases, and only 22.8% of recipients received T-cell depleting agents. The most common maintenance immunosuppressive regimen consisted of prednisone, calcineurin inhibitors and mycophenolic acid.

**Table 2:** Univariate analysis comparing KT recipients with and without solid organ cancer or lymphoma

	ALL	WITHOUT cancer	WITH cancer	p- Value
	(n=925)	(n=817)	(n=108)	
Basal characteristics				
Recipient age (years, mean ± SD)	47.9 ± 14.2	47.8 ±14.4	48.32 ± 13	0.765
Sex Female (n, %)	345 (37.2)	308 (37.6)	37 (34.3)	0.499
Caucasian race (n, %)	857 (93.3)	751 (92.5)	106 (99.1)	0.011
Arterial hypertension (n, %)	744 (81%)	663 (81.8%)	81 (75%)	0.194
Diabetes mellitus (n, %)	115 (12.5%)	106 (13%)	9 (8.3%)	0.166
Primary kidney disease: PKD vs. others (n, %)	120 (13.5)	99 (12.7)	21 (29.4)	0.054
Pre-KT cancer (n, %)	42 (4.5)	36 (4.4)	6 (5.6)	0.590
Previous transplant (n, %)	131 (14.2)	117 (14.3)	14 (13)	0.704
RRT before KT (n, %)				0.088
None	19 (2.1)	19 (2.4)	0 (0)	
HD	131 (14.2)	694 (86.9)	101 (94.4)	
PD	87 (9.6)	82 (10.3)	5 (4.7)	
KT	5 (0.6)	4 (0.5)	1 (0.9)	
Time in RRT (months, median [IQR])	22 [11–41]	22 [11–40]	23 [11–41]	0.925
Type of donor (n, %)				0.059
Standard criteria donor	550 (60.4)	476 (59.2)	74 (69.8)	
Expanded criteria donor	258 (28.4)	232 (28.9)	26 (24.5)	
Living donor	102 (11.2)	96 (11.9)	6 (5.7)	
Donor age (years, mean ± SD)	46.9 ± 16.3	47.3 ±16.3	43.9 ± 16.2	0.034
Donor sex female (n, %)	371 (42.1)	331 (42.5)	40 (38.8)	0.474
Donor Caucasian race (n, %)	793 (95.2)	703 (95.1)	90 (95.7)	0.792
**Initial immunosuppression**				
Thymoglobulin induction (n, %)	185 (22.8)	152 (21.3)	33 (34)	0.005
Calcineurin inhibitor (n, %)	788 (94.5)	695 (94.6)	93 (93.9)	0.800
Mycophenolate (n, %)	589 (64.2)	529 (65.2)	60 (56.1)	0.063
mTOR inhibitor (n, %)	21 (2.3)	20 (2.5)	1 (0.9)	0.319
**1 year afterKT immunosuppression**				
Calcineurin inhibitor (n, %)	778 (91.8)	682 (91.7)	96 (92.3)	0.824
Tacrolimus levels (ng/ml, mean ± SD)	8.2 ± 3	8.1 ± 3	9 ± 3.1	0.156
Cyclosporine levels (ng/ml, median [IQR])	212 [176–322]	214 [177–344]	208.5 [175–252]	0.678
Tacrolimus use (n, %)	470 (60.4)	422 (61.9)	48 (50)	0.026
Mycophenolic acid derivatives use (n, %)	503 (59.5)	451 (60.8)	52 (50)	0.036
mTOR inhibitor use (n, %)	54 (6.4)	51 (6.9)	3 (2.9)	0.120
**Follow-up**				
Biopsy proven acute rejection (n, %)	130 (14)	117 (14.3)	13 (12)	0.527
CMV infection (n, %)	125 (13.8)	108 (13.5)	17 (16)	0.477
BK virus (n, %)	25 (3.3)	23 (3.4)	2 (2.4)	0.616

Abbreviations: SD: Standard deviation, PKD: Polycystic kidney disease, KT: Kidney transplantation, RRT: Renal replacement therapy, HD: Hemodialysis, PD: Peritoneal dialysis, IQR: Interquartile range, mTOR: Mammalian target of rapamycin inhibitor, CMV: Citomegalovirus.

No differences were found among recipients with and without cancer in terms of age at transplantation, sex, cause of ESKD, pre-transplant malignancy, previous KT, type of renal replacement therapy (RRT), time on RRT, donor characteristics between groups, biopsy proven AR, CMV infection and BK virus. Likewise, initial and one-year treatment after transplantation with calcineurin inhibitors or mTORi showed no association with PTM development.

In the univariate analysis, recipient′s Caucasian race (92.5% vs 99.1%, p = 0.011), thymoglobulin induction (34% vs 21.3%, p = 0.005), maintenance immunosuppression with cyclosporine vs tacrolimus (55.3% vs 38.3%, p = 0.002) and treatment with mycophenolate mofetil (MMF) 1 year after-KT (60.8% vs 50%, p = 0.036) were significantly associated with the development of cancer after transplantation. (Table 2).

However, Poisson Regression analysis confirmed that only thymoglobulin induction was an independent risk factor for post-KT cancer diagnosis [Incidence Rate Ratio (IRR) 1.62 (95%CI, 1.02–2.57), p = 0.041). The association between cancer and maintenance immunosuppression with cyclosporine vs Tacrolimus or MMF was lost after adjustment for covariates (recipient age, type of donor and thymoglobulin induction). (Table 3).

**Table 3:** Multivariate analysis: poisson regression

	IRR	95% confidence interval	p- Value
Recipient age	1.014	[0.997 - 1.032]	0.112
Type of donor (living donor vs others)	0.360	[0.086 - 1.503]	0.086
Thymoglobulin induction	1.619	[1.019 - 2.571]	**0.041**
Tacrolimus vs. Cyclosporine 1 year after KT	0.731	[0.409 - 1.307]	0.291
Mycophenolate 1 year after KT	0.745	[0.427 - 1.298]	0.298

Abbreviations: IRR: Incidence rate ratio, KT: Kidney transplant.

On the other hand, the number of patients treated with thymoglobulin was similar between those who developed PTLD and solid organ cancer (36.4% vs 33.8% respectively, p = 0.823).

### Survival analysis

**Figure 5:**
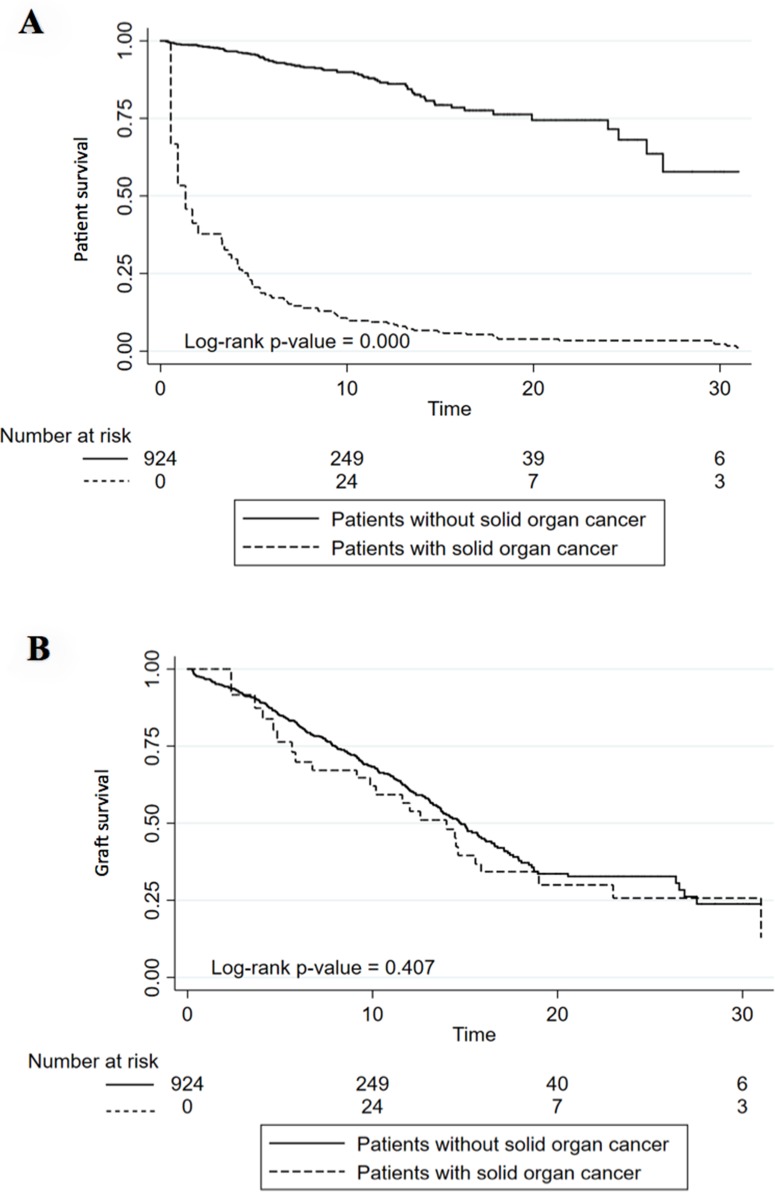
Patient (A) and Graft (B) survival in patients with and without solid organ cancer or lymphoma **(A)** Kaplan–Meier curve shows twenty-year death-censored graft survival of kidney transplantation patients with and without cancer. **(B)** Kaplan–Meier curve shows twenty-year mortality rates of kidney transplantation patients with and without cancer.

Kaplan Meier analysis showed that patients with PTM had lower survival rates compared to patients who did not develop cancer at the end of follow-up. Ten-year patient survival was 89.8% in those recipients without cancer vs 10.7% in those patients who developed cancer (p<0.001). (Figure 5A). Death-censored graft survival analysis (grafts lost not due to patient death) did not show statistically significant differences between patients with PTM and those who did not develop cancer at 10 years of follow up (62.1% vs 68.3, p<0.001). (Figure 5B).

## DISCUSSION

This retrospective study analyzed the incidence and risk factors of cancer in a cohort of 925 KT recipients. The main finding is that the true increase in cancer in KT compared with the general population occurs mostly in women. We observed an incidence of 11.7% PTM, with an overall cumulative incidence that reached 20.7% of patients at 20 years. Lung was the most common solid organ affected. Compared with the general population, the greatest increase was seen in gynecological malignancies, lung cancer and PTLD in women; and bladder, PTLD and kidney cancer in men. Cancer conferred lower patient survival and only thymoglobulin was an independent risk factor for PTM diagnosis.

The increased incidence of cancer in KT recipients is well established [4, 11, 14, 28-31, 33]. Previous series and reports have described that cumulative incidence of solid organ cancers after KT increases from 4–5% at 5 years to 10% at 10 years and to >25% after 20 years of KT, similar to our results [9-11, 34]. As expected, the incidence of cancer after KT in our cohort was also increased compared to general population with a SIR of 1.81 in women and a non-significant 1.22 in men. These rates were lower than the ones described in previous studies with an incidence of cancer among KT recipients two to four-fold higher than those age and sex-matched individuals from general population [4, 14, 28–30, 35]. Despite that apparent difference with the previously published data, our results are quite similar to those found in the European population [28, 31].

As we previously mentioned, cancer locations in KT patients differ from malignancy types occurring most frequently in general population (*e.g.,* breast, prostate and colorectal). The most affected cancer locations in Catalonia [32] and Hospital del Mar [36] are prostate, colorectal, breast and lung. When analyzing organ location affected in our cohort, PTLD, lung, prostate and bladder were the most frequent PTM, although the greatest increase in PTM incidence among KT recipients compared with the general population was seen in gynecological malignancies, lung cancer and PTLD in women; and bladder, PTLD and kidney cancer in men. Interestingly, this cancer distribution was slightly different in comparison with other PTM series and publications in which lip, thyroid and liver had relatively higher incidences [4, 35, 37–41]. Although 68% of lung cancers were in male recipients, when analyzing SIRs computed in our cohort compared to the general population in Catalonia, relative risk was more increased in women (SIR = 10.05 vs 2.22 in men). This could be explained by the large difference in lung cancer rates among men and women in the Catalan population, where men presented with significant higher rates than women (82.8 vs 17.6 per 100,000 inhabitants-year, respectively) compared to other registries where lung cancer incidence was not that different between sexes [42]. In our cohort, eight women had lung cancer: 6 of them were former or active smokers and one was diagnosed early post-transplant (probably not related to immunosuppression). Previous authors have also described a more significant increased risk of lung cancer in female transplant recipients compared to male recipients [43].

Observational studies and registries analyses have shown a great variety of PTM risk factors. Age at transplantation confers an increased absolute risk. However, whereas the absolute risk of cancer among KT recipients increases with older age, the higher relative risk of cancer seems to be greatest in younger transplant recipients, which is in part owing to the scarcity of cancer in the general population at younger ages [34, 40, 44]. Similarly, it has been described up to 20-30% higher risk of cancer for male and white ethnicity transplant recipients [4, 5, 21]. However, several studies comparing sex differences in PTM rates do not confirm this data [11, 43–45]. A recent multicenter study of 262 female kidney graft recipients in Vienna reported that 12.2% developed PTM within the first 8.4 years after KT, similar incidence to the one reported in the majority of the studies that usually include men [46]. In agreement with our findings, two studies have described a higher risk of cancer between female KT recipients than men. Kim *et al.*[43] reported a higher age-standardized SIR for all cancers in female recipients (SIR = 1.9 vs 1.6 in men). Webster *et al.*[44] found a strong likelihood that women would be diagnosed with a cancer if got a KT before the age of 45 compared to men (males vs. females <45 years HR 0.76). Probably, the role of virus-related gynecological neoplasms might influence this increased risk in women. It is far known that KT recipients have a high risk to develop virus-associated cancers such as HPV related anogenital ones. Female recipients have 14-fold increased risk of cervical cancer, up to 50-fold of vulvar cancer and up to 100-fold of anal cancer [47–50]. Epidemiological studies often suffer from underreporting of events, which typically leads to an underestimation. However, in our cohort women had been followed-up very closely and including regular gynecologic visits in addition to the routine follow-up visits due to KT. Catalan female KT recipients have up to 11 times more risk of having a gynecological cancer than general population women counterparts. This risk, together with 10-fold increase in lung cancer probably explain the higher global SIR in women female patients compared to men that we found in our cohort.

Other key risk factors for the development of cancer after-KT would be time on dialysis before transplantation, donor characteristics, having a previous KT or pre-transplant cancer history, immunosuppression treatment, biopsy proven AR and other immunological factors, smoking or alcohol use and oncogenic viruses [5, 16, 17, 19, 20, 22, 44, 51]. On the contrary, KT recipients with diabetes mellitus seem to have a 20–30% lower risk of cancer in the USA, Australia and New Zealand [5, 44]. Polycystic kidney disease (PKD) has also been related to a lower incidence of cancer compared to other causes of ESKD in KT patients [52]. We found no differences between groups in terms of recipient and donor characteristics, having or not pre-transplant cancer, previous KT or AR. Unfortunately, some data such as lifestyle (tobacco, alcohol use, etc) and oncogenic viruses like HPV and EBV were not available in our study.

The increased incidence of cancer in KT recipients is largely attributed to immunosuppression. Prior studies have focused on the relationship between PTM and different immunosuppressive agents, dose regimens and duration of the immunosuppressive therapy [3, 25, 53, 54]. The effect of each drug on cancer risk remains controversial and the increased risk of cancer may be mediated by the total burden of immunosuppression more than by the agent itself [3, 55]. However, information on dose and duration of therapy is not complete in most databases so researchers tend to investigate the relationship between PTM and selected immunosuppressive agents used for induction/maintenance therapy. Some studies have shown an association of induction immunosuppression with T cell-depleting antibodies with an increased risk of PTLD and melanoma [23, 25, 56]. In contrast, IL-2Ra induction has not been associated with significant increase of PTM [23]. In our study thymoglobulin induction was an independent risk factor for cancer. In fact, it was related to a 62% increased risk of having a new cancer diagnoses compared with those patients who did not received the drug, although it was not relate to any specific cancer location.

In terms of maintenance immunosuppression, Gallagher *et al.*[3] found no differences when analyzing overall cumulative incidence of non-skin cancer 20 years after transplantation between three different immunosuppression strategies: azathioprine and prednisolone, cyclosporine monotherapy, or cyclosporine monotherapy followed by a switch to azathioprine and prednisolone after 3 months. Nevertheless, a dose-dependent effect of cyclosporine on cancer development was demonstrated in a study using cyclosporine as maintenance immunosuppression [57] On the other hand, mTORi have previously demonstrated to inhibit rather than promote cancer in experimental models and to be associated with reduced incidence of posttransplant *de novo* malignancies in human in previous studies [26, 27, 58–60]. Recent studies and reviews [61, 62] have suggested no association between the drug itself and risk of cancer development and all-cause mortality in KT recipients. Our results showed an increased risk of cancer with cyclosporine vs tacrolimus and MMF treatment 1 year after-KT. However, this association was lost after adjustment for covariates.

All these data suggest that no immunosuppressive treatment strategies have been proved to reduce non-skin PTM risk. This entails an arduous decision for the nephrologist when choosing the best immunosuppression therapy, especially in those patients with high risk of cancer.

Additionally, based on our findings and previous literature, developing PTM entails a much lower survival rate in KT recipients compared to patients who did not develop cancer. Survival after 10 years of follow-up was 79.1%, lower than those patients without malignancy after KT. In fact, not only the survival would be poorer compared to recipients without cancer, but also compared to those individuals with cancer in general population [35, 63]. Farrugia *et al.*[7] recently described the higher malignancy-related mortality rates among KT recipients versus general population and stratified by age and gender. However, making direct comparisons between transplant recipients and general population could lead to inaccurate data, as adjustments cannot be fully made for certain types of people that are over-represented in a specific cohort. Similar findings have been described in other studies: in a Dutch kidney transplant cohort, malignancies in KT population were more aggressive and developed at a much later stage than those in patients without transplants. This led to a lower median patient survival after the diagnosis of cancer (2.7 years compared to an average survival of recipients without cancer of 8.3 years [p <0.0001]) [64]. Eventually, ANZDATA registry and other studies [10, 65] have underlined that one-third of deaths with a functioning allograft is due to cancer.

KT recipients who develop cancer represent a challenge since they require a more complex therapeutic approach. On one hand, immunosuppressive treatment tends to be minimized due to the diagnosis of malignancy albeit the possibility of rejection. And secondly, these patients may receive less aggressive cancer treatment due to comorbidities. All these elements contribute to worse prognosis for many malignancies in immunosuppressed hosts.

Cancer is a major limitation in achieving optimal outcomes in organ transplantation. Its incidence is high and it entails poorer prognoses. Further development of approaches to prevention and screening early detection of malignancy may play an important role in reducing the burden of malignancies in KT recipients. Thus, prevention of post-transplant malignancy-related morbidity and mortality must be considered a main endpoint in solid organ transplant programs [38, 39, 66–68]. Clinical guidelines recommend routine cancer screening for all KT patients, but these recommendations are mostly extrapolated from the general population [69, 70]. In fact, there is sparse evidence to support routine screening, risk factors management, and interventional therapies for KT patients [71–73].

The strengths of our study included the high validity of the cancer diagnoses based on cytological and pathological evidence, and the long duration of the follow-up that allowed us to detect the late-onset cancers, increasing the statistical power. Regarding limitations, this was a retrospective study and some relevant clinical information might be limited. Second, the comparison with the general population was taken from data reported in the literature (Catalan Registry of Cancer), which may constitute a selection bias. However, both the area and the time span of the reference population were similar to those in our cohort. Moreover, the number of patients in our cohort is not large enough to accurate the estimated risks of less common cancers. Finally, risk factors of cancers such as lifestyle, smoking and alcohol use, ultraviolet exposure, skin type, family history or oncogenic viruses such as HPV or EBV were not recorded in the database.

In summary, the increased incidence of cancer in KT when compared with the general population occurs mostly in women. Lung is the most common solid organ affected, accounting for 30% of all solid organ cancers in our cohort. Bladder, gynecological and lung cancers had the greatest SIRs compared to the general population. More importantly, thymoglobulin could be a modifiable risk factor. Our findings should stimulate research into carcinogenic mechanisms associated with organ transplantation. A greater understanding of cancer-related incidence and/or mortality risk after KT will allow clinicians to tailor modifiable risk factors such as immunosuppression. Antitumor surveillance in selected patient groups, particularly in women, and further development of prevention and screening strategies are needed to improve transplant outcomes.

## MATERIALS AND METHODS

### Patients

This retrospective cohort study used clinical and epidemiological information collected among 1038 individuals who, between 1979 and 2014, underwent KT in Hospital del Mar, Barcelona, Spain. Patients with a follow-up shorter than 90 days after KT (n = 96) and those who were lost to follow-up (n = 17) were excluded from the analysis. The final cohort consisted of 925 KT. Median time to follow-up was 8 (interquartile range (IQR) 3.26–11.29) years.

### Study variables

Recorded baseline data included recipient characteristics (age, sex, ethnicity, body mass index, cause of ESKD [categorized as diabetic nephropathy, glomerulonephritis, PKD, vascular/hypertensive disease, interstitial nephropathy, unknown or other], time on dialysis before transplant, type of RRT [categorized as pre-dialysis, hemodialysis, peritoneal dialysis or KT]), comorbidities (diabetes, coronary artery disease, peripheral vascular disease, cerebrovascular disease, previous cancer, human immunodeficiency virus, hepatitis B virus, hepatitis C virus), and transplant related factors such as the era of transplantation.

All patients with a clinical diagnosis of cancer after KT (solid organ tumors and lymphomas) were considered as cases. A cancer diagnosis required documentation of histopathological evidence. Skin tumors were excluded for the analysis.

### Statistical analysis

Continuous data were expressed as means ± standard deviation (SD) or median and IQR according to their distribution. Categorical data were expressed as percentages. Comparisons of baseline characteristics between recipients with and without cancer were made using Chi^2^ or Fisher's exact tests to analyze categorical variables, Student's T-test for continuous variables with normal distribution, and Mann–Whitney test for nonparametric variables.

Risk factors for cancer diagnosis were evaluated through Poisson Regression model. Cox proportional hazards models were used to assess death-censored graft loss, uncensored graft loss and all-cause mortality. Death-censored graft loss was considered from the transplant date to the beginning of an alternative RRT (return to dialysis o re-transplantation). For non-censored for death graft survival (uncensored graft loss) time of exposure was considered from the transplant date to the beginning of an alternative RRT or death.

Survival analysis of cancer patients and non-cancer patients was performed using Kaplan–Meier survival curves, applying the log-rank test.

Relative risks of non-skin cancers compared to the general population in Catalonia were expressed as SIRs, and these were computed using sex- and organ location. Confidence intervals were calculated using Vandenbroucke short-cut method. Cancer incidence in Catalonia was collected from the existing literature [32, 74, 75].

Statistical analysis was performed using STATA 15.0 version. A *p* value <0.05 was considered as statistically significant.

## Abbreviations

AR: Acute rejection
CKD: Chronic kidney disease
CsA: Cyclosporine
EBV: Ebstein-Barr virus
ESKD: End-stage kidney disease
HPV: Human papillomavirus
IL-2Ra: Interleukin-2 receptor alpha chain
IQR: Interquartile range
KT: Kidney transplantation
MMF: Mycophenolate mofetil
mTORi: Mammalian target of rapamycin inhibitor
PKD: Polycystic kidney disease
PTLD: Post-transplantation lymphoproliferative disorders
PTM: Post-transplant malignancies
RRT: Renal replacement therapy
SD: Standard deviation
SIR: Standardized incidence ratios
